# Size-Effect-Based Dimension Compensations in Wet Etching for Micromachined Quartz Crystal Microstructures

**DOI:** 10.3390/mi15060784

**Published:** 2024-06-14

**Authors:** Yide Dong, Guangbin Dou, Zibiao Wei, Shanshan Ji, Huihui Dai, Kaiqin Tang, Litao Sun

**Affiliations:** 1 SEU-FEI Nano-Pico Center, Key Laboratory of MEMS of Ministry of Education, Southeast University, Nanjing 210096, China; 230208145@seu.edu.cn (Y.D.); 220226059@seu.edu.cn (Z.W.); 220226387@seu.edu.cn (H.D.); 220226172@seu.edu.cn (K.T.); 2 Nanjing Jingxin Optoelectronics Research Institute, Nanjing 210096, China; ji_ss@crystron.cn

**Keywords:** quartz, MEMS, wet etching undercut, dimension compensation, microfabrication

## Abstract

Microfabrication technology with quartz crystals is gaining importance as the miniaturization of quartz MEMS devices is essential to ensure the development of portable and wearable electronics. However, until now, there have been no reports of dimension compensation for quartz device fabrication. Therefore, this paper studied the wet etching process of Z-cut quartz crystal substrates for making deep trench patterns using Au/Cr metal hard masks and proposed the first quartz fabrication dimension compensation strategy. The size effect of various sizes of hard mask patterns on the undercut developed in wet etching was experimentally investigated. Quartz wafers masked with initial vias ranging from 3 μm to 80 μm in width were etched in a buffered oxide etch solution (BOE, HF:NH_4_F = 3:2) at 80 °C for prolonged etching (>95 min). It was found that a larger hard mask width resulted in a smaller undercut, and a 30 μm difference in hard mask width would result in a 17.2% increase in undercut. In particular, the undercuts were mainly formed in the first 5 min of etching with a relatively high etching rate of 0.7 μm/min (max). Then, the etching rate decreased rapidly to 27%. Furthermore, based on the etching width compensation and etching position compensation, new solutions were proposed for quartz crystal device fabrication. And these two kinds of compensation solutions were used in the fabrication of an ultra-small quartz crystal tuning fork with a resonant frequency of 32.768 kHz. With these approaches, the actual etched size of critical parts of the device only deviated from the designed size by 0.7%. And the pattern position symmetry of the secondary lithography etching process was improved by 96.3% compared to the uncompensated one. It demonstrated significant potential for improving the fabrication accuracy of quartz crystal devices.

## 1. Introduction

Quartz crystal has become the most widely used piezoelectric material distinguished by its excellent physical and chemical properties [1,2,3,4,5]. Since Statek [6] applied Micro-Electro-Mechanical system (MEMS) process technology to the fabrication of quartz crystal devices for the first time in 1973, quartz MEMSs (QMEMSs) provided a good starting point for all kinds of sensors and actuators, such as gyroscopes [7,8,9,10], accelerometers [11,12,13,14] and oscillators [15,16,17]. Since then, wet etching in fluoride-based solutions has been one of the most fundamental QMEMS techniques used in the process of fabricating quartz devices [18].

**Figure 1:**
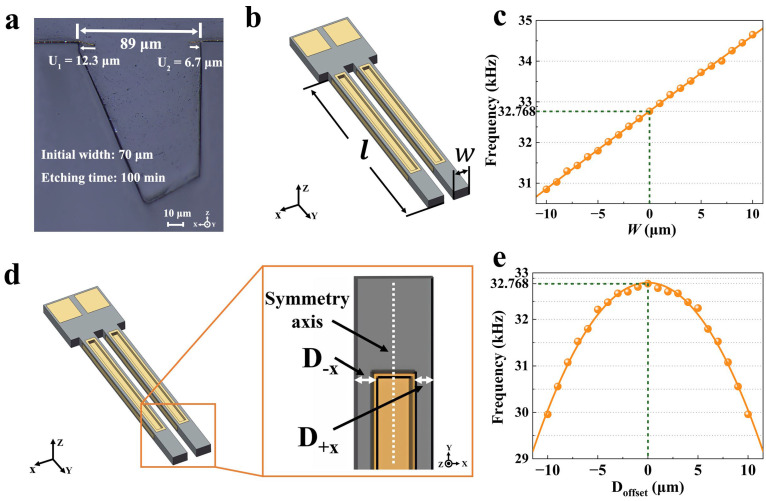
(**a**) Etching groove with an initial width of 70 μm etched in 100 min; (**b**) ultra-small quartz MEMS tuning fork resonator; (**c**) the effect of beam width on resonant frequency; (**d**) surface etching grooves of the tuning fork and its offset; (**e**) the effect of *D_offset_* on the resonant frequency.

Wet etching is advantageous when compared to dry etching due to its low cost [19,20,21]. However, undercuts between the quartz substrate and metal hard mask (Au/Cr films are the most commonly used) caused by etchants during the fabrication of the quartz microstructures became an unavoidable but undesired phenomenon [22,23,24]. As shown in Figure 1a, the undercut in the **X** direction reached 12.3 μm and 6.7 μm, respectively, for a trench with a hard mask width of 70 μm etched in 100 min. And the actual trench width reached 83.4 μm with an increase of 19.14% compared to the designed size, which changed the actual size of the device and had a serious impact on the precision of the fabrication. These variations in dimensions could even affect the functionality of the microstructure.

Figure 1b shows the ultra-small quartz MEMS tuning fork oscillator in our studies.

The device was fabricated on a Z-cut quartz wafer. As shown in Formula (1) [25], the frequency of the tuning fork was significantly determined by the form size.

(1) $$f\propto A\frac{w}{l^{2}}$$

The coefficient *A* was determined by the properties of the quartz itself. For a quartz wafer with a fixed cut angle, the coefficient can be considered constant. *w* and *l* are the width and length of tuning fork beams, respectively. Figure 1c shows the effect of the beam width on frequency. It was obvious that the frequency was markable increased by the beam width. Even a 1 μm variation in beam width could change the frequency by approximately 14,000 ppm. Therefore, even small processing errors had a significant impact on the performance of devices. Actually, in mass production, metal deposition and lasers are always used together to adjust the frequency. It took about 2 s per chip to adjust frequency deviation from −10,000 ppm to −1000 ppm by using laser beams. If the initial frequency of the tuning fork can be controlled at −5000 ppm or even lower by improving the manufacturing accuracy, the processing time would take about 0.5~1 s, which would be an almost 100% increase in efficiency. Thus, improving the manufacturing accuracy of tuning forks is an essential requirement.

Furthermore, due to the anisotropic etching of the quartz crystal, the undercut affected not only the apparent shape but also the position symmetry of the device, which also markedly influenced the characteristics of the quartz devices. As shown in Figure 1d, the surface etching grooves were the critical parts which were used to reduce the resonant impedance of the quartz tuning fork [26,27,28]. However, the offset of the etching grooves caused by anisotropic etching significantly affected the performance of it. In Formula (2), $D_{offset}$ describes the displacement of the surface etching groove caused by anisotropy etching. $D_{+x}$ and $D_{-x}$ are the distances of the surface etching groove from **−X** and **+X**, respectively.

(2) $$D_{offset}=D_{+x}-D_{-x}$$

As shown in Figure 1e, the resonant impedance of the tuning fork showed a clear quadratic relationship with $D_{offset}$. And even small position offsets could influence the resonant impedance markedly. An offset of 5 μm resulted in a frequency deviation of nearly 21,300 ppm (700 Hz), which was much worse than the market demand for that of less than 8 ppm (2.6 Hz). Thus, position fabrication errors caused by anisotropic undercuts had an extremely noticeable effect on the functionalities of devices. Therefore, it was particularly important and necessary to consider the undercut and dimensional compensation in process design.

However, the anisotropic properties of the quartz crystal made the etching result more complex and difficult to predict because the etching rate of individual crystal planes defining the structure and their interdependence were decisive parameters in defining the exact geometrical shape of the microstructures. No systematic studies of the anisotropic etching of quartz crystal had been reported until 1987; Ueda et al. [29] demonstrated the etching anisotropy of quartz by using 21 kinds of cut angles to predict the cross-sectional shapes of micromechanical devices made of quartz wafers. Hedlund [30] and Liang [22] first considered the undercut in their research. Tellier et al. [31] used a kinematic model to simulate the quartz etching results. But they focused more on collecting reliable data on the crystallographic dependence of the etching depth of multi-cut-angle quartz planes rather than the Z-cut plane undercut, which was the most widely used cut type. More regrettably, despite the fact that the quartz MEMS has been developed for decades, no compensation strategies have been reported so far, but they have been intensively studied in silicon fabrication [32]. However, nowadays, quartz devices are becoming smaller and smaller in size to meet the requirements of portable and wearable devices, which places a higher requirement on the fabrication precision of quartz crystal devices.

Therefore, in this paper, the laws between undercut and hard mask width were first explored, based on which two novel dimensional compensation strategies were proposed, which were employed to fabricate an ultra-small quartz tuning fork resonator with 32.768 kHz. The actual etched size of critical parts of the device only deviated from the designed size by 0.7%, and the pattern position symmetry of the secondary lithography etching process was improved by 96.3% compared to the uncompensated one. The method showed great potential for improving the fabrication accuracy of quartz crystal devices.

## 2. Materials and Methods

### 2.1. Pattern Design

**Figure 2:**
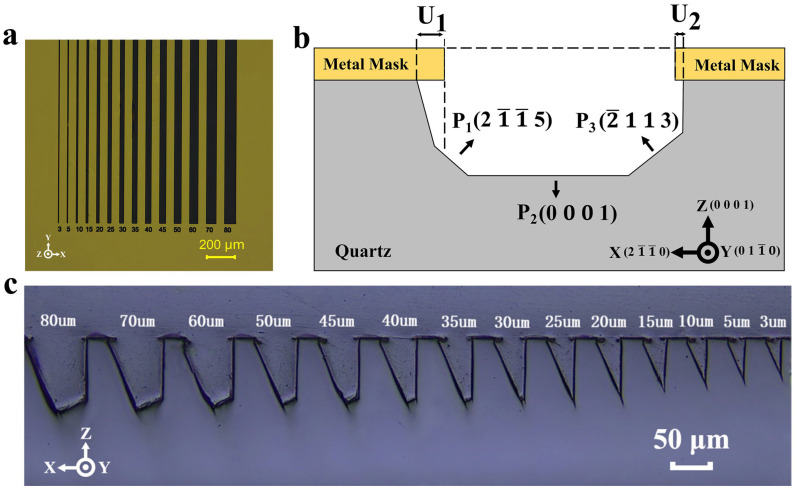
(**a**) Long-strip patterns of the metal hard mask widened from 3 μm to 80 μm; (**b**) proposed etching plane (**P_1_**, **P_2_**, **P_3_**) of a typical bilateral cross-section of the etched grooves; (**c**) optical image of the cross-sections of the 14 grooves after 100 min etching.

If the device size is large enough, the initial hard mask width can be changed enough to match the device size. And the difference in hard mask width is meaningless for etching ultra-small quartz devices. Therefore, this work focused on fourteen ultra-small hard mask widths (<100 μm), which are more valuable for improving the etching precision of ultra-small resonant devices. The initial long-strip pattern widths ranged from 3 μm to 80 μm and were divided into 3 groups, A (3 μm, 5 μm), B (from 10 μm to 45 μm in intervals of 5 μm), and C (from 50 μm to 80 μm in intervals of 10 μm) (Figure 2a). All the long strips were 1 mm in length and 50 μm apart, pointing to the **Y** direction. And the samples were etched with different etching times.

### 2.2. Sample Preparation

The 3-inch Z-cut quartz crystal wafers used in this work had a thickness of 300 μm and met the conditions of Q value ≥ 2.4 million, inclusion density class Ia, and etching channel density ≤ 30 bars/cm^2^, and they were manufactured by Crystron Technologies Inc., China. The quartz wafers were washed in piranha solution (H_2_SO_4_:H_2_O_2_ = 3:1) at 90 °C for 10 min. A set of Au (310 nm) and Cr (60 nm) films were sputtered onto quartz wafers as metal mask layers, patterning the metal films with the designed photoresist mask. And the coated wafer was etched in mixture etchants with 49 wt% HF and 40% wt% NH_4_F (HF:NH_4_F = 2:3) at 80 ± 1 °C. The first sample was taken after 3 min of etching. After that, every 5 min for 120 min, a piece of the sample was taken out. And the ultra-small tuning fork was fabricated based on the same quality quartz wafer with a thickness of 100 μm.

### 2.3. Profile Definition

Although Hedlund [30] and Liang [22] have already reported the definition of a Z-cut quartz plane etched profile morphology, this definition was only suitable for macroscopic descriptions of etching results with large hard mask widths. For the purpose of this work, a novel and concise definition was proposed to satisfy the characterization of small-hard-mask-width etching profiles. As shown in Figure 2b, a complete bilateral etched groove is demonstrated in this work, which is different from previous work where only half of the etched profile was defined. And angles were not imported into the definition, which would greatly simplify the characterization of the etching profile. Instead, three planes were defined (**P_1_**$\left(2\bar{1}\bar{1}5\right)$, **P_2_**(0001), **P_3_**$\left(\bar{2}113\right)$), which made it easier to present the evolution of the etching profiles. In particular, this work made full use of undercuts to design a new fabrication compensation process; therefore, the depths of the undercuts were exclusively represented by **U_1_** and **U_2_**, respectively. This definition of the profile focused the variables on characterizing the complete evolution of the etching groove itself, rather than the individual etching rate, which would facilitate the hard mask width to enable the design of a number of novel processes in practical engineering applications.

### 2.4. Measurement

After etching, quartz wafers were cut vertically to the etching slots and the etched morphologies were observed and measured by optical microscopy (Olympus, Suzhou, China) and a profiler (Dektak, Billerica, MA, USA). In order to succinctly visualize the etching results, the average values of the three samples were collected at the same time and plotted in this paper. Photographs of the fourteen grooves are shown in Figure 2c. The fabricated quartz device tuning fork was measured by an impedance analyzer for electrical parameters (Tonghui, Suzhou, China).

## 3. Results and Discussion

### 3.1. Evolution of Bilateral Etching Profiles

**Figure 3:**
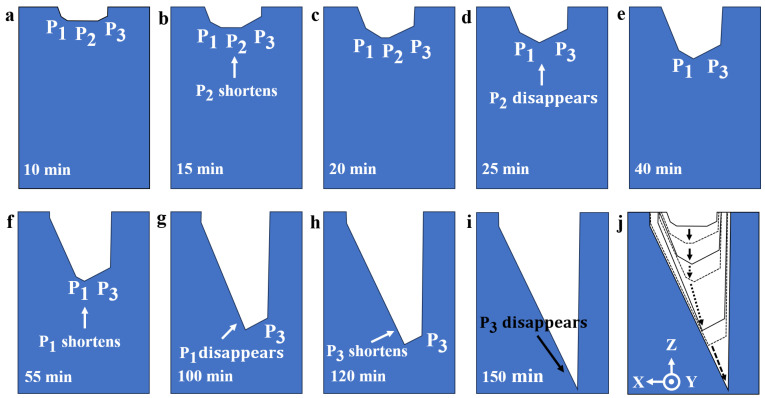
The etching evolution of grooves with 45 μm initial width in 150 min. (**a**) The three etching planes appeared; (**b**) **P_2_** shrunk gradually; (**c**) **P_2_** shrunk gradually; (**d**) **P_2_** disappeared. (**e**) **P_1_** shrunk gradually; (**f**) **P_1_** shrunk gradually; (**g**) **P_1_** disappeared; (**h**) **P_3_** shrunk gradually; (**i**) **P_3_** disappeared; (**j**) the complete evolution.

As far as we know, nobody has obtained complete etched results with bilateral etching profiles when etching Z-cut quartz crystal wafers. In this study, we observed the evolution of the complete morphology of Z-cut quartz crystals. As a representative, we schematically reproduced the etching evolution of grooves with 45 μm initial widths in 150 min, as shown in Figure 3. It was evident that, to start with, there were three etching planes in the slot, which were defined as **P_1_**, **P_2_**, **P_3_** (Figure 2b and Figure 3a). At shallower etching depths, the etching grooves were almost symmetrical, which meant that there was no large difference in etching depth between the individual crystal planes (Figure 3b). As the etching depth increased, a large difference in etching depth between the different crystal planes emerged. At 25 min, the **P_1_** and **P_3_** platforms increased in length and the **P_2_** platform was the first to begin shrinking and eventually disappeared (Figure 3c,d). Thereafter, **P_1_** shrunk and disappeared at approximately 100 min (Figure 3e–g). The disappearance of **P_1_** reduced the etchant concentration in the etching grooves, which affected the etching results of undercuts. In particular, **P_1_** disappeared very early for small widths, such as 5 μm, whose **P_1_** disappeared almost in 40 min. Then, **P_3_** decreased and finally disappeared at 150 min (Figure 3h,i). When **P_3_** disappeared, an asymmetric sharp groove was obtained (Figure 3i). Figure 3j shows the complete evolution in an aggregated way. It was obvious that although the etching rate in the **X** direction was much lower than that in the **Z** direction, the tiny dissolution of the **X**-direction crystal planes still resulted in the actual etched width being larger than the initial width, which would reduce the actual fabrication accuracy. The variation of the three etching planes played an important role in the later analysis of the undercut etching results.

### 3.2. Effect of the Hard Mask Width on the **U_1_**

In past studies, few researchers have specifically focused on the effects of undercuts in quartz crystal wet etching. In fact, the manufacturing errors caused by undercuts can hardly be ignored in real fabrication. Therefore, the study of undercuts is of great practical engineering significance. Only when the etching law of undercuts is familiarized and understood can engineers consider dimension compensations more accurately when designing devices. In this work, the behaviors of undercuts in quartz wet etching were experimentally summarized.

#### 3.2.1. Size Effect on **U_1_**

**Figure 4:**
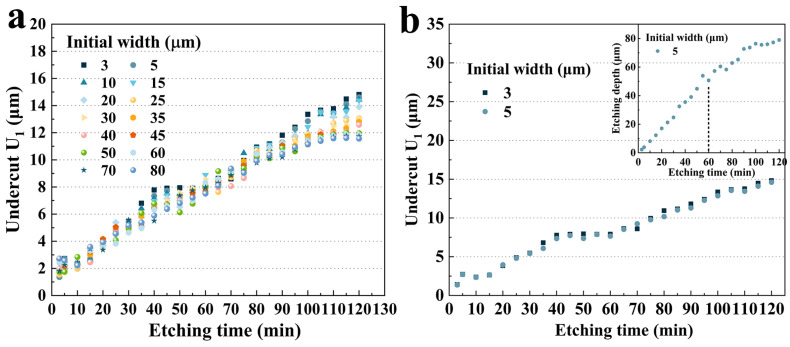
(**a**) **U_1_** with different initial widths versus etching time; (**b**) the **U_1_** of group A versus etching time and the etching depth of 5 μm versus etching time.

As shown in Figure 4a, at different hard mask widths, **U_1_** tended to increase almost linearly with an increase in etching time. The maximum depth even increased to 14.81 μm in 120 min. It can be seen that the curves could be divided into three stages. Firstly, from 3 to 40 min, the slopes of the etching depth curves were almost the same. The growth trend of undercuts with different hard mask widths was the same. And then, from 40 to 60 min, there seemed to be a plateau, where some of the curves flattened out and the undercut **U_1_** increased insignificantly as the time increased. At the third stage, from 60 to 120 min, remarkable differences in undercut growth appeared for different hard mask widths. It was clear that curves with larger hard mask widths grew more slowly (group C), while curves with smaller ones (group A) continued to climb linearly and dramatically.

In more detail, as shown in Figure 4b, for group A, there was little difference in **U_1_** between the two widths in 120 min, which was due to the fact that the difference between the initial widths of the two curves was too small. Interestingly, it can be clearly seen that the curve is divided into three stages. And between 40 and 60 min, the undercut leveled off with etching time. During this period, the more easily etched crystal plane **P_1_** already disappeared (at 10 min), and **P_3_** continually decreased and disappeared at 30 min. Therefore, according to the etching kinetics, it can be speculated that less etchant infused into the etching grooves on the quartz crystal led to the **X**-direction etching rate being reduced and flattened. Subsequently, after 60 min, the **U_1_** continued to increase with etching time. This was due to the fact that the groove etching rate slowed down and much more etchant etched the **X**-plane, as shown in the inset. Furthermore, a huge undercut appeared during the plateau stage, which resulted in the actual etched width becoming much larger than the hard mask width. Therefore, much more etchant infused into the etched grooves, which also made the **U_1_** continue to increase.

**Figure 5:**
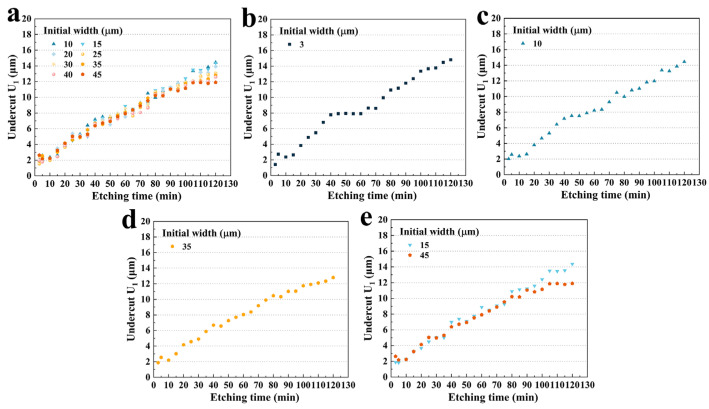
(**a**) **U_1_** for group B versus etching time; (**b**) **U_1_** with an initial width of 3 μm versus etching time; (**c**) **U_1_** with an initial width of 10 μm versus etching time; (**d**) **U_1_** with an initial width of 35 μm versus etching time. (**e**) The **U_1_** with initial widths of 15 μm and 45 μm versus etching time.

As shown in Figure 5a, for group B, **U_1_** increased approximately linearly with the etching time. But the slow-growing platforms were less noticeable. The plateau period from 40 to 60 min gradually tapered off as the hard mask width grew. This phenomenon is more obvious in Figure 5b–d. It was obvious that from 3 μm to 10 μm, the plateau became less pronounced. At an initial width of 35 μm, the plateau almost disappeared, while for 80 μm, no visible plateau could be seen during this period. This might be caused by the fact that the crystal plane **P_3_** of wider hard mask widths took a longer etching time to disappear, allowing the etchant to continuously etch the **Z**-plane. And due to the etching kinetics, the etchant could continue to enter the etching grooves and etch the **X** direction at a relatively uniform etching rate.

Interestingly, for a fixed etching time, a larger hard mask width resulted in a smaller **U_1_**. Figure 5e shows the etching results of 15 μm and 45 μm. It was obvious that after 95 min, the etching rate decreased. According to etching profile evolution, this was caused by the disappearance of the **P_1_** platform at this time. And at 120 min, there was a 17.2% increase in the **U_1_** for a width of 15 μm (14.75 μm) compared to that for 45 μm (14.375 μm). However, this increase was not significant in the middle of the etching process. For example, at 45 min, the **U_1_** for an initial width of 15 μm (7.381 μm) was only 9.03% higher than that for an initial width of 45 μm (6.713 μm), which was almost half of that at 120 min.

**Figure 6:**
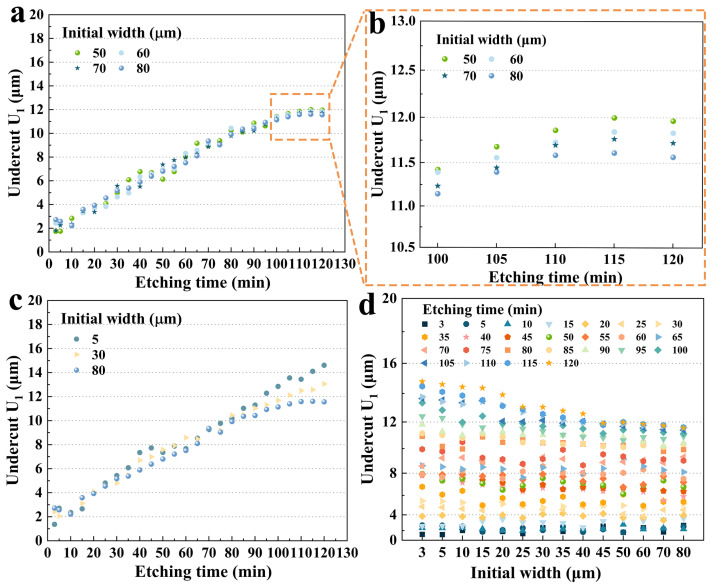
(**a**) **U_1_** for group C versus etching time; (**b**) **U_1_** of group C versus etching time from 100 to 120 min; (**c**) **U_1_** for selected data sets versus etching time; (**d**) the undercut **U_1_** with different hard mask widths for the fixed etching time.

Similarly, these results also applied to group C, as shown in Figure 6a,b. It was evident that the growth of **U_1_** tended to slow down after 100 min, which also coincided with the disappearance of **P_1_**. Furthermore, **U_1_** with different hard mask widths had a more subtle difference compared to group B. At 120 min, the **U_1_** for a hard mask width of 80 μm (11.566 μm) was only 3.33% lower than the undercut for that of 50 μm (11.965 μm).

Figure 6c shows three typical etching results from the three groups’ data sets with large hard mask width differences. It was clear that the size effect on the **U_1_** was negligible in the first 35 min. From 40 to 90 min, the **U_1_** showed extremely small and irregular size-effect phenomena but continued to appear with etching time. After 95 min, a significant difference in the size of the undercut appeared. In particular, at 120 min, 80 μm and 30 μm undercuts were reduced by 20.8% and 10.6%, respectively, compared to 5 μm. This phenomenon is more obvious in Figure 6d. It demonstrated that the size effect on **U_1_** was apparent and remarkable for a fixed etching time of 65 min and created a difference of maximum 3.3 μm, which had a significant impact on the design process of quartz crystal devices in practical engineering.

#### 3.2.2. Size Effect on Etching Rate **V_u1_**

**Figure 7:**
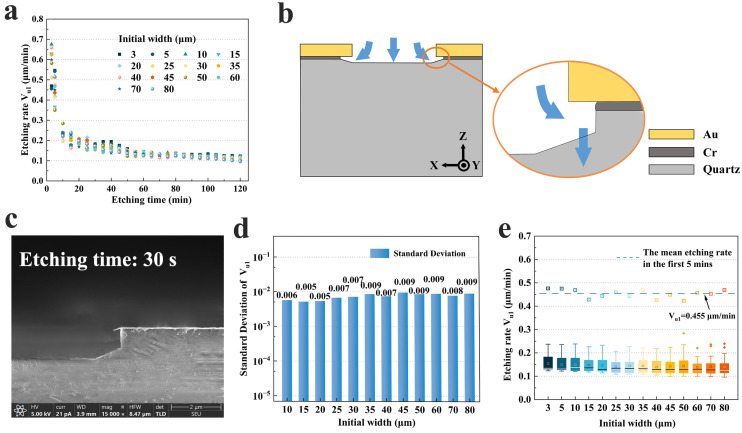
(**a**) The etching rate **V_u1_** versus etching time for different initial widths; (**b**) schematic diagram of etchant-etched metal layer and formed undercut; (**c**) SEM image of a hard mask width of 40 μm etched for 30 s; (**d**) the standard deviation of the etching rate from 75 to 120 min for representative widths; (**e**) box plots for etching grooves from 10 to 120 min.

Based on the etching depth data, the etching rates for **U_1_** were calculated. Figure 7a shows the etching rate **V_u1_** versus time for different hard mask widths. It was very interesting to note that the etching rate **V_u1_** was extremely high during the early etching period. The highest etching rate reached 0.675 μm/min (10 μm) in the first 3 min, while the lowest etching rate reached 0.456 μm/min (5 μm). However, **V_u1_** decreased rapidly thereafter and eventually stabilized at around 0.1 μm/min. This indicated that the undercut did not form slowly and gradually, but almost as quickly in the first 5 min. This might be due to the fact that at the beginning of the etching, the etching solvent reacted with the **Z**-plane, creating the shallow etching depth and dissolving part of the Cr-metal layer, which acted as an adhesion layer. And then the etchant dissolved the quartz under the Cr layer immediately, as shown in Figure 7b. This phenomenon was also confirmed in Figure 7c, which shows the SEM image of the hard mask width of 40 μm etched for 30 s. It was obvious that even though it was etched for only 30 s, regular crystal surfaces had been formed under the undercut.

Subsequently, due to the slow rate of crystal etching in the **X** direction, the vast majority of the etchant continued to etch the **Z**-plane, resulting in a reduced etching rate of the undercut. The etching rate for different hard mask widths also maintained almost the same trend with etching time. Figure 7d illustrates the standard deviation of the etching rate from 75 to 120 min for representative widths. It was obvious that for a fixed width, the etching rate **V_u1_** did not change considerably after 75 min, and the standard deviations were all less than 0.01. This result is more obvious in Figure 7e. There was a high concentration of **V_u1_** for each hard mask width over a period of 10 min to 120 min, whereas the etching rate in the first 5 min was plotted separately, and the average etching rate in the first 5 min (0.455 μm/min) was about 3.7 times that after it stabilized. It is worth noting that the average etching rate varies slightly for different initial widths. A wider hard mask width had a slightly lower **V_u1_** than the thinner one. As discussed previously, the size effect had a stronger impact on the etching rate in the later stages of etching (>90 min).

**Figure 8:**
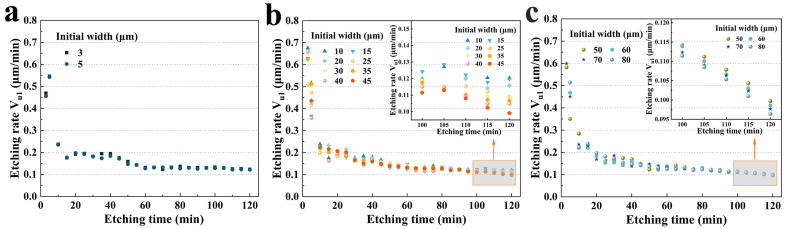
(**a**) **V_u1_** versus etching time for group A; (**b**) **V_u1_** versus etching time for group B; (**c**) **V_u1_** for group C versus etching time.

In more detail, as shown in Figure 8a, for group A, the etching rate (0.469 μm/min, 3 μm; 0.465 μm/min, 5 μm) decreased to 0.123 μm/min for 3 μm, and 0.122 μm/min for 5 μm, which was a nearly 3.8-fold decrease. However, there was no significant difference in the etching rate because the initial widths of the two were not considerably different. Figure 8b illustrates the **V_u1_** for group B. Similarly, in the first 5 min, the etching rate was almost 3.7 (40 μm) to 6.8 (10 μm) times that of 120 min. More interestingly, as shown in the inset of Figure 8b, after 100 min, the size effect was much more obvious. It was evident that an increase in the initial width decreased the etching rate **V_u1_**. The etching rate of 45 μm (0.09 μm/min) was 21.2% lower than that of 10 μm (0.12 μm/min). Group C exhibited more pronounced trends, as shown in Figure 8c and its inset.

### 3.3. Effect of the Hard Mask Width on the **U_2_**

#### 3.3.1. Size Effect on **U_2_**

**Figure 9:**
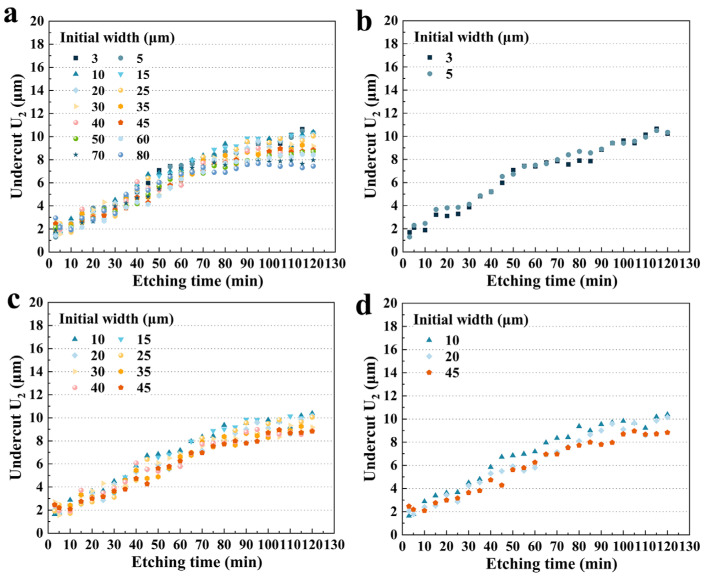
(**a**) The undercut **U_2_** versus etching time for different initial widths; (**b**) the undercut **U_2_** versus etching time for group A; (**c**) the undercut **U_2_** versus etching time for group B; (**d**) the undercut **U_2_** versus etching time for three representative initial widths of 10 μm, 20 μm, and 45 μm.

The undercut **U_2_** exhibited a different trend to **U_1_**, as shown in Figure 9a. Although **U_2_** with different initial widths increased with etching time, variations in **U_2_** tended to flatten when the etching time exceeded 80 min and maxed out at 10.386 μm (10 μm) in 120 min. This meant that if the device needed to be etched for more than around 80 min, there would be no need for extensive dimensional compensation in the **–X** direction. Figure 9b illustrates the undercut of group A with etching time. The size effect was not obvious. An inflection point occurred at 60 min, and the change in **U_2_** became gradual. As determined earlier, at this time, the etching depth had reached its limit, and the etchant began to release in the **−X**-plane. At the later stage of the etching, there was no difference between the two grooves with similar **U_1_** values, which may be due to the small difference in initial width.

However, group B reflected a different trend, as shown in Figure 9c. The curve could be divided into two stages. Before 80 min, **U_2_** increased almost linearly with etching time. Subsequently, the growth slowed down. This was due to the disappearance of the **P_1_** around 80 min. Interestingly, the size effect for group B was pronounced throughout the whole etching process. This phenomenon was even more evident in Figure 9d, which shows three representative data sets. It was clear that throughout the etching process, the curves with smaller hard mask widths were above the curves with larger ones. At 120 min, 45 μm **U_2_** was reduced by 14.9% compared to 10 μm. Thus, for a fixed etching time, the larger the initial width, the smaller the **U_2_**.

**Figure 10:**
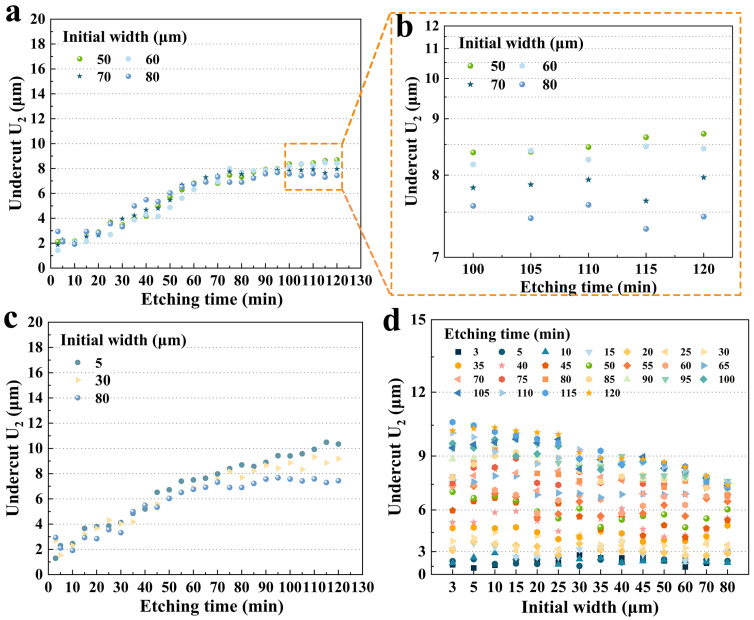
(**a**) The undercut **U_2_** for group C versus etching time; (**b**) **U_2_** for group C versus etching time after 100 min; (**c**) **U_2_** versus etching time for three representative initial widths of 5 μm, 30 μm, and 80 μm; (**d**) **U_2_** versus initial widths at fixed etching time.

This trend also applied to group C, as shown in Figure 10a. After 85 min, a smaller initial width resulted in a larger **U_2_**. At 120 min, the undercut of 80 μm was reduced by 14.4% compared to that of 50 μm (Figure 10b). The difference was due to the fact that for different hard mask widths, the **P_1_** disappeared at different times. However, in the early stage of etching, especially before 60 min, the size effect on **U_2_** was not significant, which was different from group B.

Figure 10c shows three sets of data with large differences in initial widths. Overall, for a fixed etching time, a larger initial width resulted in a smaller **U_2_**. Over the period of 100 to 120 min, the average undercut depth of 5 μm was 10.5% and 24.9% higher than that of 30 μm and 80 μm, respectively. And this difference in etching became remarkable after 80 min. It is more comprehensively illustrated in Figure 10d, which shows the **U_2_** of various hard mask widths for fixed etching times. It was clear that around 80 min, the size effect became more pronounced. According to the etching profile evolution, the **P_3_** platform was becoming smaller and disappearing during this period and the etching depth of the groove was almost reaching the limit. Therefore, it could be hypothesized that less of the etchant was drawn into the etching groove to participate in the reaction according to the etching kinetics, and the corresponding etching rate in the **X** direction slowed down and tended to stabilize. Furthermore, different initial widths would result in different times for the **P_3_** to shrink, which would lead to various times for the reduction in the etchant in etching grooves and ultimately result in different etching depths of **U_2_**.

#### 3.3.2. Size Effect on Etching Rate **V_u2_**

**Figure 11:**
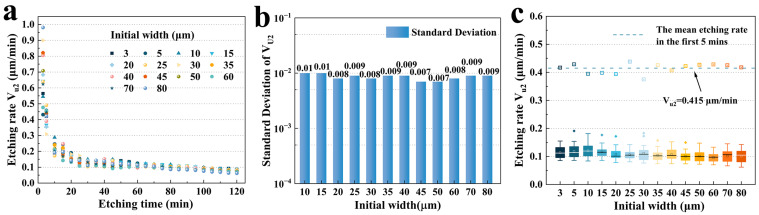
(**a**) The etching rate **V_u2_** versus etching time for different initial widths; (**b**) standard deviation of the **V_u2_** from 80 min to 120 min for representative widths; (**c**) box plot of each groove from 20 min to 120 min.

The etching rate **V_u2_** of **U_2_** exhibited a similar pattern to **U_1_**, as shown in Figure 11a. It was obvious that the etching rate **V_u2_** was also quite high at the beginning of the etching process, reaching a maximum of 0.98 μm/min (80 μm) and a minimum of 0.432 μm/min (5 μm) in the first 5 min. After 5 min, **U_2_** dropped rapidly and continuously to about 0.06 μm/min, which was almost half of that of **U_2_**. However, **U_2_** plateaued in about 30 min, while **U_1_** took almost 60 min. Therefore, it meant that the undercut was still formed very rapidly when etching began. And it also indicated that the etching rate of the **+X**-plane was approximately twice that of the **−X**-plane. In the middle stage of etching, the size effect was not obvious. However, after 80 min, the etching rate apparently received the effect of the initial width, which was consistent with the patterns of **U_1_**. Figure 11b demonstrates the standard deviation of the etching rate from 80 to 120 min. For a fixed initial width, the etching rate varied extremely slightly over this time period, with standard deviations all less than 0.01, which was also consistent with the results of the former analysis of **U_2_**. Similarly, Figure 11c shows the box plot of each groove from 20 to 120 min. During this period, the centrality of **V_u2_** was also high for a fixed initial width. The **V_u2_** for the first 5 min was plotted individually, with an average etching rate of 0.421 μm/min, which was nearly identical to **V_u1_** and approximately 6.9 times the stabilized etching rate.

**Figure 12:**
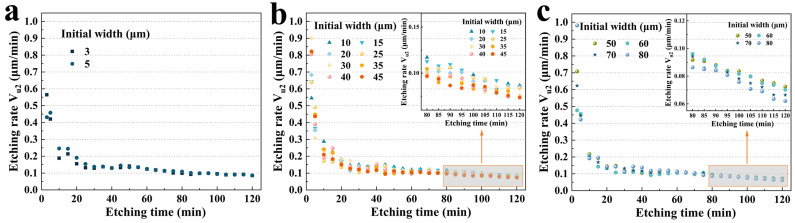
(**a**) **V_u2_** versus etching time for group A; (**b**) **V_u2_** versus etching time for group B; (**c**) **V_u2_** versus etching time for group C.

Figure 12a–c show details of the variation in **V_u2_** with etching time for groups A, B, and C, respectively. It could be seen that there was not too much difference between the three groups. For groups B and C, the initial width had a slight effect on the etching rate after 85 min (inset of Figure 12b,c). While this difference is small enough for most quartz devices, with the development of ultra-small quartz devices, these small differences still have significant impacts on the processing accuracy of fabrication. Therefore, these results of the undercuts are of great importance for dimensional compensation in actual device fabrication.

### 3.4. Size-Effect Dimensional Compensation for Quartz MEMS Tuning Fork

As mentioned earlier, undercuts were a non-negligible factor in the processing of quartz crystal devices. In particular, with the popularization of portable and wearable devices, higher demands are being placed on the fabrication accuracy of ultra-small quartz devices. Therefore, dimensional compensation has become an essential part of the design and manufacturing of quartz crystal devices. The etching results described above for different initial widths can be used for dimensional compensation in the fabrication of quartz devices, which can be extremely effective in improving processing accuracy. And the accurate prediction of the compensation value can effectively reduce the difference between the designed and etched structure. This work used the above etching results to devise a dimensional compensation solution, which was divided into etching width compensation and etching position compensation. And these compensation solutions were used to finely fabricate a kind of ultra-small MEMS quartz crystal tuning fork (TF) resonator with a resonance frequency of 32.768 kHz. The 100 μm thick quartz wafer was used to fabricate the tuning fork in this work.

#### 3.4.1. Etching Width Dimension Compensation

Quartz crystal tuning forks have become one of the most widely used quartz resonator devices, which are used in a wide range of applications such as clock resonators, accelerometers, transducers [33], quartz-enhanced photoacoustic spectroscopy, and so on. As illustrated in Formula (1), the frequency of the tuning fork was determined by the width and length of the tuning fork beams. In fact, since frequency was an inversely proportional function of l, the frequency was no longer sensitive to variations in length at a beam size smaller than 1500 μm. Instead, the beam width w had an extremely high effect on the frequency of the tuning fork (Figure 1c). A variation in width of 1 μm could cause the frequency to change by approximately 450 Hz (13,732 ppm). So, this work mainly focused on the effect of width on the tuning fork.

**Figure 13:**
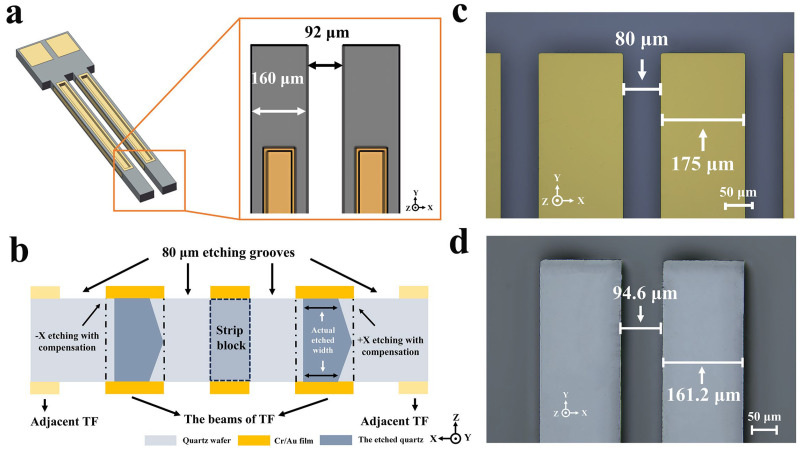
(**a**) The diagram of the fabricated tuning fork and its target beam size; (**b**) schematic of the etching width dimension compensation; (**c**) designed pattern size; (**d**) actual etched size.

Figure 13a shows the diagram of the fabricated tuning fork and its target beam width size. The target width of the beam was 160 μm and the target gap between the two beams was 92 μm. Etching width compensation could effectively improve the actual etching accuracy. Figure 13b illustrates the principle of etching width dimensional compensation. The core idea of etching width compensation is to compensate for the depth of the undercut to achieve the designed dimensions based on the etching laws of the chosen initial etching width.

In more detail, based on the effect of initial width on the undercut, an etching groove with an initial width of 80 μm was selected to split the gap between the two beams. Since a 100 μm thick quartz wafer was selected to fabricate the tuning fork, based on previous etching results, it was predicted that double-sided etching would take 70 min if the devices were etched by using an initial width of 80 μm. According to the previous analysis, it could be predicted that undercuts of 8.5 μm (**U_1_** = 8.5 μm **V_u1_** = 0.12 μm/min) in the **+X** direction and 6.5 μm (**U_2_** = 6.5 μm **V_u2_** = 0.093 μm/min) in the **−X** direction were expected. Thus, 8.5 μm and 6.5 μm of etching width dimensional compensation were used for the **+X** and **−X** directions, respectively, as shown in Figure 13c. Therefore, based on the target size, the TF beam pattern was designed as 175 μm. Figure 13d shows the etching results after 70 min of etching with the same BOE etchant. For accurate measurements, the metal mask layers of Au/Cr on the surface were removed. The actual etched TF beam width was 161.2 μm and the actual etched gap width was 93.6 μm. The etching width compensation provided a manufacturing error of only 0.7%.

Therefore, the compensation value could be predicted more accurately based on the undercut with different initial widths, which greatly improved the accuracy of manufacturing. It also helped the researchers design devices with a basis in mind. In particular, it was possible to make predictions for ultra-small critical widths, which could have a very positive effect in practical engineering.

#### 3.4.2. Etching Position Compensation

**Figure 14:**
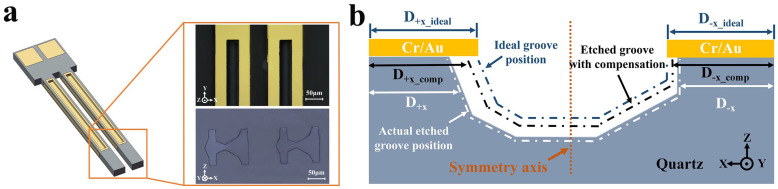
(**a**) The diagram of the fabricated TF, the typical surface etching grooves, and their cross-section; (**b**) schematic of the principle of etching position compensation.

As mentioned previously, tuning forks are often used as resonant devices to make various types of sensors or actuators. And for tuning fork resonant devices, the most important electrical characteristic is the resonant impedance. The lower the resonant impedance, the better the performance of the device. And surface etching grooves on the tuning fork beams fabricated by secondary lithography and etching are always used to reduce the resonant impedance, which can significantly enhance the performance of the tuning fork. Figure 14a shows the typical surface etching grooves and their cross-section.

However, according to the previous analysis, the undercut would have different etching characteristics in the **+X** and **−X** directions, which could result in an offset between the actual surface etching groove and the desired one. Even worse, this offset had an extremely pronounced effect on the tuning fork characteristics, as mentioned in Figure 1e. Therefore, it became critical to correct the position of the etching groove by means of etching position compensation. Figure 14b shows the principle of etching position compensation. The core idea was to predict the offset caused by the anisotropic undercut based on the etching laws for the selected initial width and to reserve the corresponding position compensation in the design of the pattern mask. Obviously, the position of the etching groove was significantly offset in the **+X** direction relative to the position in the ideal state due to anisotropic etching. Nevertheless, as represented by the black dashed line, it was possible to predict the offset and correct the position of the etching grooves by using the etching laws of undercuts at different initial widths, which can significantly reduce the impact of offset.

**Figure 15:**
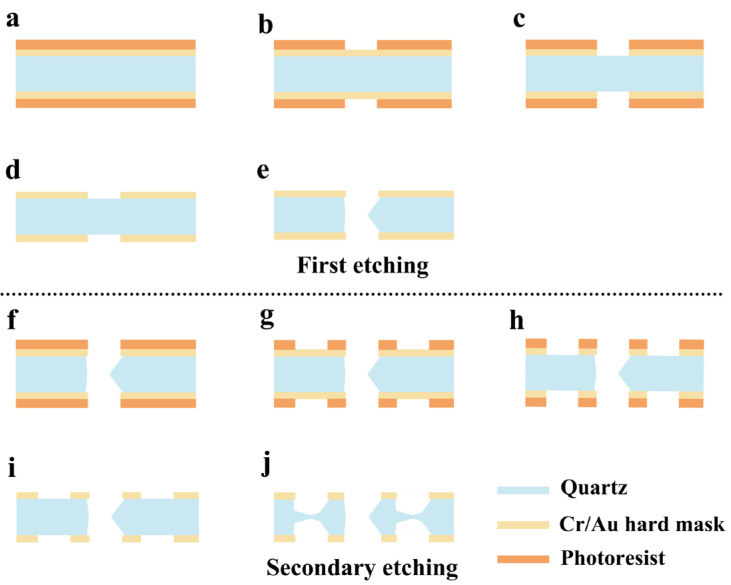
(**a**) The quartz wafer with metal hard mask and photoresist; (**b**) lithography and development of device shape; (**c**) pattern of the Cr/Au hard mask; (**d**) remove photoresist; (**e**) etch device shape in etchant; (**f**) spray photoresist; (**g**) secondary lithography and development of surface etching grooves; (**h**) pattern of the Cr/Au hard mask; (**i**) remove photoresist; (**j**) etch surface etching grooves in etchant.

Figure 15 illustrates the process flow diagram for the fabrication of surface etching grooves. It was clear that surface etching grooves needed to be made by two photolithographic processes. Firstly, the tuning fork shape was created by the first photolithography and etching process. Subsequently, the surface etching grooves were fabricated by a secondary patterning and etching process. It is worth noting that the tuning fork beams continued to be etched while the surface etching grooves were being etched. Therefore, the consequences of the two etching processes had to be considered.

**Figure 16:**
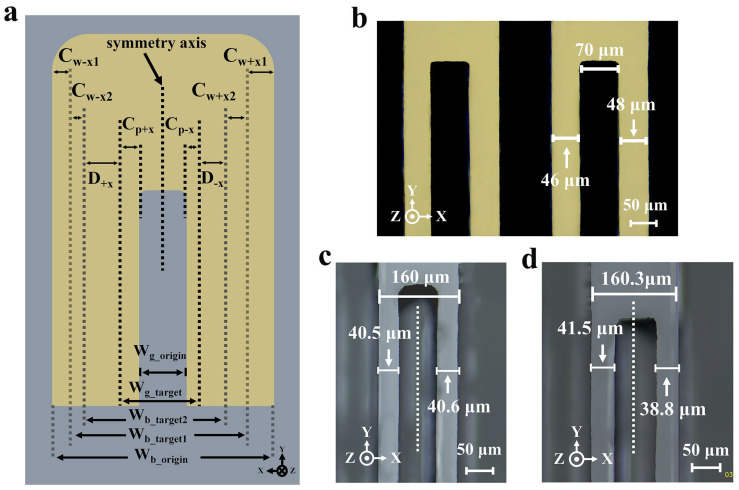
(**a**) The schematic of the etching dimension compensation with two rounds of etching width compensation and one round of etching position compensation; (**b**) the designed pattern with 2 μm etching position compensation after the first etching stage; (**c**) the actual etching result with position compensation; (**d**) the actual etching result without position compensation.

Figure 16a illustrates the schematic of the etching compensation, including two rounds of etching width compensation and one round of etching position compensation. $W_{b-origin}$, $W_{b-target1}$, and $W_{b-target2}$ are the initial beam width, the target beam width of the first etching stage, and the target beam width of the second etching stage, respectively. $C_{w+x1}$ and $C_{w+x2}$ are the first and second etching width compensation stages of the tuning fork beam in the **+X** direction. $C_{w-x1}$ and $C_{w-x2}$ are the first and second width compensation stages of beams in the **−X** direction. $C_{p+x}$ and $C_{p+x}$ are the position compensation for the surface etching grooves in the **X** direction. $D_{+x}$ and $D_{-x}$ are the distance between the actual boundary of the surface etching groove and the actual boundary of the tuning fork beam, respectively. $D_{offset}$($D_{offset}=D_{+x}-D_{-x}$) is the actual position offset of the surface etching grooves relative to the beams.

In this work, 70 μm was chosen as the initial width of the surface etching groove, with a target etching depth of 40 μm. Based on the above etching results, the expected etching time was 30 min. We predicted that the width of the beam would continue to shrink by about 4.8 μm during these 30 min. In detail, the undercut $C_{w+x2}$ was 3 μm in the **+X** direction (**V_u1_** = 0.1 μm/min) and $C_{w-x2}$ was 1.8 μm in the **−X** direction (**V_u2_** = 0.06 μm/min). A width error of nearly 5 μm had an impact of over 20,000 ppm on the final frequency of the device. Therefore, in order to achieve the target size, in the first pattern step, the etching width compensation was utilized to allow for the etching deviation generated by the second lithography. In this work, the initial device’s hard mask pattern was reserved for 15 μm compensation for the first etching step and 5 μm for the second etching step. Eventually, the width of the initial beam hard mask was designed to be 180 μm. Meanwhile, in terms of etching position compensation, based on the previous analysis, for the surface etching groove with a 70 μm initial width, the undercut $C_{p+x}$ in the **+X** direction at 30 min was in the region of linear growth and was predicted to be 5.7 μm (**V_u1_** = 0.19 μm/min). At the same time, the undercut $C_{p-x}$ in the **−X** direction was hypothesized to be 3 μm (**V_u2_** = 0.1 μm/min). Thus, it caused an etching position offset of around 2.7 μm in the **+X** direction, which resulted in a large frequency difference of about 11,000 ppm (360 Hz).

At the same time, in particular, the offset of the symmetry axis of the beam itself caused by the anisotropic undercut in the **X** direction ($C_{w+x2}$ and $C_{w-x2}$) during the second etching process also needed to be taken into account when designing the compensation of the etching position, as shown in Formula (3).

(3) $$D_{b-offset}=C_{W+x2}-C_{w-x2}$$

Therefore, synthesizing the above analysis, it could be predicted that the position offset of the final surface groove conformed to Formula (4).

(4) $$D_{offset}=D_{g-offset}-D_{b-offset}$$

Thus, as shown in Figure 16b, after the first etching step, a 2 μm etching offset was reserved for the second photolithography and etching step. Figure 16c and Figure 16d compare the etching results with etching position compensation and without compensation. For accurate measurements, the metal mask layers of Au/Cr on the surface were removed. It was noteworthy that the etching position compensation resulted in an effective reduction of the etching position offset to 0.1 μm. The symmetry improved by more than 96.3% compared to the uncompensated one with 2.7 μm offset.

**Figure 17:**
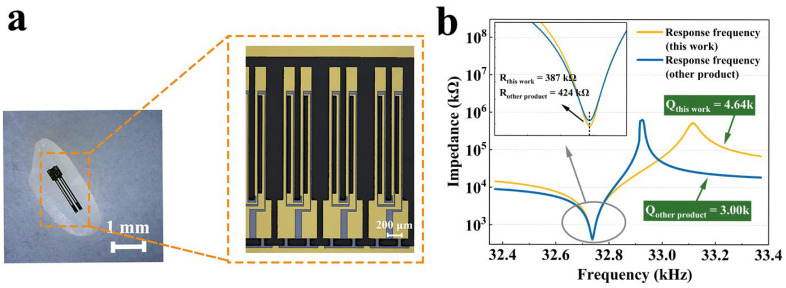
(**a**) The fabricated ultra-small tuning fork device placed on a rice grain; (**b**) the testing result of the tuning fork compared with a certain company’s product.

Finally, Figure 17a shows the ultra-small tuning fork device fabricated in this work. For size illustration, the device was placed next to a rice grain. The tuning fork was tested by an impedance analyzer at normal atmospheric pressure, and Figure 17b illustrates the testing results. It is worth noting that with these meticulous compensation strategies, it was even possible to use the metal deposition and laser frequency tuning processes, which greatly enhanced production efficiency. Furthermore, a tuning fork manufactured by a certain company was used for comparison. The impedance of this working tuning fork was 8.7% lower and the quality factor Q was 54.6% higher than that of the comparison one.

In summary, these two kinds of dimension compensation solutions with different initial widths could greatly reduce the discrepancy between the design targets and the actual etching results and effectively improve the fabrication accuracy of quartz crystal devices.

## 4. Conclusions

In this work, we focused on the size effect of the undercut on the Z-cut quartz crystal in wet etching, experimentally summarized the etching patterns, and came up with two kinds of dimensional compensation strategies to fabricate a kind of ultra-small quartz crystal tuning fork resonator. In this process, the complete bilateral etching morphology was defined, and the etching depth and rate of undercut were used to characterize the size effect on the etching results. It was found that the undercut **U_1_** in the **+X** direction for different initial widths grew almost linearly with etching time. The initial width began to have a significant effect on the etching results after 95 min of etching. The larger the initial width, the smaller the **U_1_**. The undercut **U_2_** in the **−X** direction showed different laws. During the first 80 min, **U_2_** increased approximately linearly with etching time, but after 80 min, the increase rate became slower. A larger initial line width also resulted in a smaller **U_2_** after 100 min. Interestingly, by calculating the etching rate of the undercut, we found that the undercut was formed almost in the first 5 min with a relatively high etching rate. After that, the etching rate began to increase rapidly from 15% to 27%. And the average etching rate of the undercut in the **+X** direction was almost twice as high as that in the **−X** direction. Afterwards, these etching laws were employed to develop two types of dimensional compensation solutions, etching width compensation and etching position compensation. And an ultra-compact quartz resonator with a frequency of 32.768 kHz was successfully fabricated by using the compensation solutions. The actual manufacturing error of key parts of the device was only 0.7%. The pattern symmetry of secondary etching improved by more than 96.3% compared to the uncompensated one. It confirmed a great potential in enhancing the manufacturing accuracy of quartz crystal devices. Furthermore, summarizing the etching results into better adapted mathematical models would be the future direction of this work.

## Data Availability

All data generated or used during the study appear in the submitted article.
